# Supplementary material for: Comparative genomic analysis of three geographical isolates from China reveals high genetic stability of Plutella xylostella granulovirus
Source: PLoS One. 2021 Jan 14;16(1):e0243143. doi: 10.1371/journal.pone.0243143 (PMC7808651; doi:10.1371/journal.pone.0243143)
Supplement: S1 Table — (DOCX) [file pone.0243143.s001.docx]

**S1 Table: Mutation frequency of eight PlxyGV isolates relative to the PlxyGV-W genome sequence in coding, noncoding and *hr* regions** **(×10^-3^)**

| **Isolates** | **Coding region** | | | | **Noncoding region** | | | | ***hrs*** | | | |  |
| --- | --- | --- | --- | --- | --- | --- | --- | --- | --- | --- | --- | --- | --- |
|  | **mu** | | **rate** | | **mu** | | **rate** | | **mu** | | **rate** | |  |
| PlxyGV-B | | 73 | | 0.82 | | 4 | | 0.84 | | 254 | | 53.29 | |
| PlxyGV-C | | 287 | | 3.20 | | 64 | | 13.43 | | 369 | | 54.54 | |
| PlxyGV-J | | 320 | | 3.58 | | 46 | | 9.65 | | 380 | | 56.16 | |
| PlxyGV-K | | 295 | | 3.30 | | 40 | | 8.39 | | 363 | | 53.65 | |
| PlxyGV-M | | 282 | | 3.15 | | 65 | | 13.64 | | 364 | | 53.80 | |
| PlxyGV-SA | | 1058 | | 11.83 | | 174 | | 36.51 | | 654 | | 96.66 | |
| PlxyGV-T | | 267 | | 2.99 | | 65 | | 13.64 | | 364 | | 53.80 | |
| PlxyGV-Wn | | 268 | | 3.00 | | 55 | | 11.54 | | 405 | | 59.86 | |
