# Supplementary material for: Comparative genomic analysis of three geographical isolates from China reveals high genetic stability of Plutella xylostella granulovirus
Source: PLoS One. 2021 Jan 14;16(1):e0243143. doi: 10.1371/journal.pone.0243143 (PMC7808651; doi:10.1371/journal.pone.0243143)
Supplement: S2 Table — (DOCX) [file pone.0243143.s002.docx]

**S2 Table. The transcription start sites and polyadenylation sites of PlxyGV specific genes**

| ORF | Position | aa |  | | Promotor^①^ | | | | Terminator^②^ | | |
| --- | --- | --- | --- | --- | --- | --- | --- | --- | --- | --- | --- |
|  |  |  | TSS | TATA box | | A(A/T)CGT(G/T) /CGTGC | CAGT/CAAT/CATT | G/A/TTAAG | PAS | AATAAA | ATTAAA |
| 1 | 175 〉375 | 66 | -347 |  | | -384, -212, -44 | -309, -168, -114, -66 |  | 386 | 327, 367 |  |
| 3 | 604 〉921 | 105 | -210 |  | |  | -71, -21 | -13 | 1259(501) | 1294(536) | 1248(490) |
| 5 | 1685〈 2080 | 131 | -214 |  | | -55 | -50, -132, -182 | -73 | 760 | 753, 769 |  |
| 19 | 11818〈12120 | 100 | -120, -82 | -114 | |  | -123 | -13 | 18 | 0 |  |
| 22^③^ | 14602 〉15789 | 399 | -80 |  | | -42 | -161 |  | 76 | 9, 62 |  |
| 27 | 20537 〉20776 | 79 | -877, -455, -66 | -172 | | -787, -382, -218, -106, -347, -104 | -758, -360,-263, -367, -354, -330, -300, -156, -753, -651, -311, -248, -55 |  | 157 |  | 131 |
| 33 | 27819〈 28112 | 97 | -447 |  | | -41 | -445, -404, -382, -364, -294, ①-214, -160, -275, -130, -70 |  | 44 | 26, 61 |  |
| 58 | 46913〈 47074 | 53 | -363 | -416 | |  | -320, -238, -211, -127, -85, -344, -109, -185, -143 | -91, -29 | 286 (35) | 253 (2) |  |
| 81 | 63521 〉63844 | 107 | -473 | -419, -14 | | -493, -282 | -412, -363, -256 |  | 148 | 96, 148 |  |
| 105^④^ | 86745〈 87953 | 402 | -255 | -51 | | -129 | -112, -82 |  | 49 | 28 |  |
| 108 | 90368〈 91213 | 281 | 600 | -340 | | -559 | -524, -207, -127, -115, -33, -23, -275, -248, -157 |  | 246 | 217? |  |
| 111 | 94237 〉94815 | 192 | -712 (-366) |  | | -142 | -287, -623, -463, -370, -278, -673, -608, -453, -173, -167, |  | 2 | -11 |  |
| 119 | 99751 〉100179 | 142 | -110 | -40 | |  | -9 |  | 16 | 0 |  |

1. The numbers in the columns represent the positions of the promoter elements relative to the initiation codon ATG of the indicated ORFs and the upstream ORF (the number in the bracket).
2. The numbers in the columns represent positions of the transcription terminator elements relative to the stop codons of the indicated ORFs and the ORFs downstream (the numbers in the brackets).
3. The TSS identified locates at +180 position relative to the start ATG of the large ORF ().
4. The TSS identified locates at +310 position relative to the start ATG of the large ORF ().
