# Supplementary material for: Comparative genomic analysis of three geographical isolates from China reveals high genetic stability of Plutella xylostella granulovirus
Source: PLoS One. 2021 Jan 14;16(1):e0243143. doi: 10.1371/journal.pone.0243143 (PMC7808651; doi:10.1371/journal.pone.0243143)
Supplement: Attachment — Submitted filename: Comments of reviewers.docx [file pone.0243143.s003.docx]

The current study is related to Comparative genomic analysis of three geographical isolates from China reveals high genetic stability of Plutella xylostella granulovirus. They sequenced and comparatively analyzed to investigate genetic stability and diversity of PlxyGV，PlxyGV-W, PlxyGV-B. By alignment of the genome sequences of nine PlxyGV isolates sequenced up to date, the author found that PlxyGV genome is fairly stable in nature.

Some aspects should be addressed to improve this manuscript.

1. PlxyGV-W and PlxyGV-B demonstrate higher insecticidal activity than PlxyGV-Wn for P. xylotella larvae. The LC50 of PlxyGV-Wn is about two times of the ones of the other two virus isolates while there is no significant difference between PlxyGV-W and PlxyGV-B. Could you analyze the reasons for different insecticidal virulence of the three GV isolates from the perspective of insect virus genome?
2. By alignment of the genome sequences of nine PlxyGV isolates sequenced, the author found that PlxyGV genome is fairly stable in nature. What is the enlightenment and significance of your current work for the next step in the development of recombinant viruses or insecticides with higher insecticidal virulence against Plutella xylostella?
