# Supplementary material for: Comparative genomic analysis of three geographical isolates from China reveals high genetic stability of Plutella xylostella granulovirus
Source: PLoS One. 2021 Jan 14;16(1):e0243143. doi: 10.1371/journal.pone.0243143 (PMC7808651; doi:10.1371/journal.pone.0243143)
Supplement: Attachment — Submitted filename: Response to additional requirements.docx [file pone.0243143.s004.docx]

Response to additional requirements

1. Please ensure that your manuscript meets PLOS ONE's style requirements, including those for file naming.

Answer: We have reformatted the manuscript following the guidelines.

2. We note that you have stated that you will provide repository information for your data at acceptance. Should your manuscript be accepted for publication, we will hold it until you provide the relevant accession numbers or DOIs necessary to access your data. If you wish to make changes to your Data Availability statement, please describe these changes in your cover letter and we will update your Data Availability statement to reflect the information you provide.

Answer: The accession numbers of the genome sequences of three PlxyGV isolates are included in the text (Line 122).

3. We note that you have included the phrase “data not shown” in your manuscript. Unfortunately, this does not meet our data sharing requirements. PLOS does not permit references to inaccessible data. We require that authors provide all relevant data within the paper, Supporting Information files, or in an acceptable, public repository. Please add a citation to support this phrase or upload the data that corresponds with these findings to a stable repository (such as Figshare or Dryad) and provide and URLs, DOIs, or accession numbers that may be used to access these data. Or, if the data are not a core part of the research being presented in your study, we ask that you remove the phrase that refers to these data.

Answer: All the phase “data not shown” in the manuscript have been removed.
